# Astrocytic mitochondrial frataxin—A promising target for ischemic brain injury

**DOI:** 10.1111/cns.14068

**Published:** 2022-12-22

**Authors:** Rimi Hazra, Enrico M. Novelli, Xiaoming Hu

**Affiliations:** ^1^ Pittsburgh Heart, Lung, and Blood Vascular Medicine Institute, Department of Medicine University of Pittsburgh Pittsburgh Pennsylvania USA; ^2^ Center of Cerebrovascular Disease Research, Department of Neurology University of Pittsburgh School of Medicine Pittsburgh Pennsylvania USA

**Keywords:** astrocytes, frataxin, ischemia, mitochondria

## Abstract

In the ischemic brain, hypoxia leads to mitochondrial dysfunction, insufficient energy production, and astrocyte activation. Yet, most studies investigating mitochondrial dysfunction in cerebral ischemia have focused exclusively on neurons. This review will highlight the importance of the morphological, molecular, and functional heterogeneity of astrocytes in their role in brain injuries and explore how activated astrocytes exhibit calcium imbalance, reactive oxygen species overproduction, and apoptosis. In addition, special focus will be given to the role of the mitochondrial protein frataxin in activated astrocytes during ischemia and its putative role in the pharmacological management of cerebral ischemia.

## Graphical abstract

Astrocytes interconnect cerebral microvessels with neurons. Mitochondrial function within the astrocytes regulates neuronal response during cerebrovascular ischemia. Astrocytic calcium influx may reduce frataxin (FXN) expression, thereby contributing to mitochondrial dysfunction. Targeted induction of astrocytic FXN may be an attractive therapeutic strategy for ischemic brain injury.

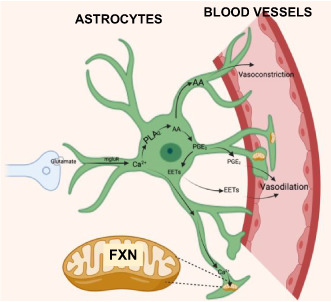

## 1 INTRODUCTION

Brain function is critically supported by an intimate anatomical and chemical relationship among the components of the neurovascular unit, namely glial cells (astrocytes, oligodendrocytes, and microglia), vascular cells (pericytes, endothelial cells, and vascular smooth muscle cells), and the basal lamina matrix.^1^ Astrocytes are the most common type of glial cells (20%–40%) within the central nervous system (CNS) and provide a critical bridge between neurons and blood supply through their unique cytoarchitectural featuring presynaptic processes and end‐feet processes that surround the cerebral vasculature.^2^, ^3^ Astrocytes serve several crucial functions, ranging from metabolic and structural support of the brain to regulation of synaptogenesis and synaptic transmission.^4^, ^5^ Specifically, astrocytes control fluid movement between the intracellular and extracellular space, protect neurons against oxidative stress, regulate energy metabolism and blood flow, and modulate neuronal activity through the expression of cytokines, growth factors, and transporters,^6^, ^7^ thereby maintaining cerebral homeostasis.

Ischemic brain injuries are global health problems with an estimated mortality of 2.7 million per year worldwide.^8^ Alterations in astrocyte signaling, metabolism, and architecture during cerebral ischemia determine the extent of brain injury and can either support or inhibit repair. Astrocytes residing in the white matter are characterized by the expression of the intermediate filament protein glial fibrillary acidic protein (GFAP), which alters calcium signaling during ischemic brain injury.^9^ Reactive astrocytes are often classified as neurotoxic (type A1), promoting neuropathogenesis and cognitive impairment, or neuroprotective (type A2).^10^ Recent studies have demonstrated that astrocytes have unique metabolic characteristics that are important during ischemia. Relative to neurons, astrocytes have higher levels of antioxidant molecules and reactive oxygen species (ROS)‐detoxifying enzymes, including glutathione, heme‐oxygenase 1, glutathione peroxidase, glutathione S‐transferase, catalase, and thioredoxin reductase,^5^, ^11^, ^12^, ^13^ likely providing astrocytes greater resistance to cellular damage induced by the prooxidant compounds hydrogen peroxide, nitric oxide, peroxynitrites, and 6‐hydroxydopamine.^12^, ^14^, ^15^ Astrocytic perisynaptic processes express various receptors for neurotransmitters, transporters, ion channels, cytokines, and growth factors.^16^, ^17^ Among these, glutamate receptors and transporters act as sensors for neuronal glutamatergic neurotransmission. Moreover, astrocytic end‐feet express glucose transporters and the aquaporin 4 water channel at their luminal surface.^16^ The glutamate‐sensing ability of astrocytes is important for their role in responding to and preventing excitotoxicity in ischemic conditions.

The majority of astrocyte functions are attributed to astrocytic mitochondria. Although mitochondria are primarily known for their role as a glucose‐derived ATP generator, they also regulate Ca^2+^ signals, coordinate metabolism, and determine cell survival/death.^18^, ^19^ Metabolically, astrocytes are highly oxidative and possess a large number of mitochondria.^20^, ^21^ Frataxin (FXN) is a mitochondrial protein present in tissues with a high metabolic rate, such as the brain, heart, liver, and kidney.^22^ The exact function of FXN has not been fully elucidated; however, it is known to play a role in iron homeostasis by acting as a chaperone during the formation of Fe‐S clusters, which are cofactors that facilitate normal enzymatic functions and regulate oxidative phosphorylation.^23^ Moreover, FXN functions as an iron storage protein during conditions characterized by iron overload.^24^ In high‐iron conditions, FXN may serve a vital antioxidant role by attenuating ROS production.^25^ In this review, we summarize the role of astrocyte mitochondria in the pathology of ischemic brain injury, with a specific emphasis on how mitochondrial dysfunction, bioenergetic defects, altered mitochondrial dynamics, and cerebral blood flow regulation impact the brain during ischemia. We also examine how the astrocytic mitochondrial protein FXN is a possible pharmacologic target for regulating the response to ischemia.

### 1.1 The role of astrocyte mitochondria and the potential contribution of FXN in metabolic response during ischemia

#### 1.1.1 Astrocytic mitochondrial responses to ischemia

Although astrocytes are more resistant to ischemia than neurons, ischemia initiates metabolic cascades in astrocytes leading to dysfunction and apoptosis. During ischemia, elevated mitochondrial Ca^2+^ activates several tricarboxylic acid (TCA) cycle dehydrogenases that induce ROS generation within the astrocytes.^26^ ROS oxidize mitochondrial lipids, sulfhydryl groups, and iron sulfur complexes that are required for respiratory enzyme function, thus impairing mitochondrial oxidative phosphorylation.^27^, ^28^, ^29^ Further, intramitochondrial calcium accumulation through mitochondrial permeability transition pore (MPTP) dissipates the mitochondrial membrane potential, resulting in antioxidant pathway failure and increased ROS production.^30^, ^31^, ^32^, ^33^, ^34^ The following osmotic swelling of mitochondrial cristae and subsequent release of cytochrome c and nicotinamide adenine dinucleotide hydrogen (NADH) into the cytoplasm initiate apoptosis signaling cascades within astrocytes.^35^, ^36^, ^37^, ^38^

When astrocytes are functioning properly, they protect neurons from excitotoxicity, an important contributor to neuronal cell death during ischemia. Astrocytes modulate ischemia‐induced neuronal excitotoxicity by alleviating excessive glutamate in the extracellular space through glutamate uptake by glutamate transporters, such as glutamate–aspartate transporter (GLAST) and glutamate trasporter‐1 (GLT‐1).^39^, ^40^, ^41^ However, ischemia‐induced neuronal glutamate release may negatively impact mitochondrial function in astrocytes. During ischemic conditions in vitro and transient oxygen and glucose deprivation (OGD, 30 min) in hippocampal slices, astrocytes undergo mitochondrial remodeling. Transient OGD decreases mitochondrial length and results in a nearly 50% loss of mitochondria from astrocyte processes. The primary drivers of these mitochondrial changes are pathological activation of glutamate transport and increased astrocytic calcium.^42^ Similarly, another study demonstrated that OGD exposure in astrocyte–neuronal cocultures resulted in astrocyte mitochondria with rounder morphology and fewer filament‐like structures and intramitochondrial connections.^34^, ^40^ Together, these in vitro studies suggest a link between ischemic brain injury and astrocyte mitochondrial dysfunction. In an in vivo study, a transgenic mouse line with an astrocyte‐specific reporter was used to assess morphological changes in astrocytic mitochondria following transient global cerebral ischemia induced by bilateral common carotid artery occlusion.^43^ Astrocyte mitochondria showed signs of dysfunction, including increased spherical morphology, decreased tubular mitochondria, and increased fusion in the ischemic brain. During reperfusion, neurons release high levels of glutamate, leading to an increase in cytosolic calcium in astrocytes and cell death through mitochondrial dysfunction.^44^, ^45^, ^46^ Glutamate plays a pivotal role in the dynamic process of astrocyte mitochondrial dysfunction, as activation of glutamate transport is necessary and sufficient to cause mitochondrial arrest within astrocyte processes.^47^, ^48^ The evidence suggests that glutamate released by neurons in response to ischemic injury may be a master regulator of astrocytic mitochondrial dynamics and functions and that mitochondrial function in astrocytes is compromised during ischemic injury. Excess glutamate release results in Ca^2+^ overload in mitochondria that uncouples electron transfer from ATP synthesis causing impaired energy metabolism and increased free radical production leading to cell death.^49^ Further research is required to fully understand how mitochondrial dysfunction alters astrocyte metabolism and function during ischemia. Nevertheless, protecting astrocytic mitochondria from damage and dysfunction may be a promising therapeutic strategy to preserve the neuroprotective role of astrocytes in ischemia and prevent ischemic damage.

#### 1.1.2 Involvement of FXN in astrocytic response

Mitochondrial FXN plays a key role in iron homeostasis and metabolism in neurons and astrocytes. Several studies of Friedreich ataxia, which features FXN deficiency, have shown differential electron transport chain activities in the absence of FXN.^50^ FXN downregulation causes mitochondrial dysfunction, reduced ATP production, oxidative stress, free radical accumulation, and consequent cell death. Indeed, upon loss of iron homeostasis, free mitochondrial iron interacts with oxygen molecules to form ROS.^51^ Compared to controls, FXN‐deficient dorsal root ganglion neurons had higher concentrations of free intracellular calcium, greater activation of caspase 3 and calpains, altered calcium‐mediated signaling pathways, and increased neurite degeneration and apoptotic cell death.^52^ Moreover, FXN‐deficient astrocytes had lower expression of several DNA mismatch repair enzymes and the antioxidant enzymes superoxide dismutase (SOD) and glutathione peroxidase (Gpx1), resulting in greater sensitivity to oxidative stress.^45^ Finally, FXN knockdown increased the production of ROS in primary mouse astrocytes.^44^ These studies provide evidence that FXN plays a crucial antioxidative role in the mitochondria of both neurons and astrocytes.

Recently, mitochondrial exchange between neurons and astrocytes has emerged as a potential regulatory pathway for maintaining neuronal viability and intracellular energetics. One study demonstrated that damaged mitochondria from retinal ganglion cell axons are transferred to adjacent astrocytes for subsequent degradation.^53^ Interestingly, in experimental ischemia, astrocytes release functional extracellular mitochondria through a calcium‐dependent mechanism.^54^ These astrocytic mitochondria contribute to neuroprotection by enhanced O‐GlcNAcylation, a posttranslational modification that attaches O‐linked β‐N‐acetylglucosamine to serine and/or threonine side chains of mitochondrial proteins to improve mitochondrial function.^55^ Notably, iron suppresses O‐GlcNAcylation in adipocytes.^56^ Thus, mitochondrial iron accumulation in astrocytes due to FXN dysregulation may regulate mitochondrial exchange. Advancing our understanding of ischemia‐induced astrocytic mitochondrial dysfunction due to FXN deregulation may allow the identification of novel pathways that contribute to ischemic brain injury.

### 1.2 Cerebral blood flow regulation by astrocytic mitochondria and the prospective role of FXN during ischemic injury

#### 1.2.1 Astrocytic mitochondria and cerebral blood flow

The neurovascular system is composed of neurons, blood vessels, and astrocytes. While the specific cell types responsible for regulating cerebral blood flow remain unknown, mounting evidence points to the involvement of astrocytes in this process. Ca^2+^ is an important regulator of cerebral blood flow, and Ca^2+^ homeostasis is primarily modulated by mitochondria. Therefore, astrocytic mitochondrial Ca^2+^ signaling may play a vital role in regulating cerebral blood flow in response to functional hyperemia.

Upon neuronal activation, astrocytes mediate the neurovascular response, which results in a coordinated change in cerebral blood flow. Glutamate released at the synapse acts on glutamate receptors and is taken up by glutamate transporters on astrocyte processes. Glutamate uptake initiates an intracellular calcium signal that is propagated to the astrocytic end‐feet and stimulates the release of vasoactive factors onto cerebral blood vessels. Vasoactive factor release dilates the blood vessels and consequently increases blood flow to meet increased metabolic demands.^57^, ^58^, ^59^, ^60^ Studies have shown that astrocytic Ca^2+^ oscillations both precede^58^, ^61^ and follow vasodilation of cerebral arterioles.^62^ However, there is a several second time delay between propagating astrocytic Ca^2+^ signals and the vasodilation response, suggesting that there is another mechanism driving the rapid blood flow changes typically seen in vivo.^62^, ^63^, ^64^, ^65^ More recent studies have confirmed the presence of stimulus‐evoked rapid calcium signals specifically within astrocyte processes and vascular end‐feet that are fast enough to cause blood vessel diameter changes in vivo.^66^, ^67^ Vasoactive factors, including nitric oxide (NO), prostaglandins, arachidonic acid metabolites, and adenosine, are released in response to astrocytic end‐feet calcium signals and mediate the functional blood flow responses. Studies in cortical brain slices using two‐photon calcium imaging in astrocyte end‐feet have shown both dilatation and constriction of adjacent arterioles during ischemia.^60^, ^63^ Changes in the diameter of the arteriolar lumen were inhibited by blocking phospholipase A2, an enzyme that causes the release of arachidonic acid (AA) from membrane lipids, and by blocking the conversion of AA into its metabolite 20‐hydroxyeicosatetraenoic acid (20‐HETE), which is a potent vasoconstrictor. Astrocyte end‐feet dilated cortical arterioles in response to transient Ca^2+^ via an AA‐dependent mechanism in vivo.^59^ AA is converted into prostaglandin E2 (PGE2) via cyclooxygenase‐1 (COX‐1), an enzyme expressed in astrocytic end‐feet. Inhibition of COX‐1 also blocked Ca^2+^‐mediated arteriole dilatation.^68^ Moreover, calcium imaging in astrocytes from hippocampal and neocortical slices revealed that high oxygen content (95% O_2_, 5% CO_2_) leads to Ca^2+^‐evoked blood vessel constriction, whereas low oxygen content (20% O_2_, 5% CO_2_) reverses constriction and induces dilatation. The blood oxygen content likely mediates extracellular lactate concentrations, thereby impacting AA metabolite levels.^69^ However, the mechanism by which oxygen modulates neurovascular coupling remains unknown and has yet to be observed in vivo. Overall, studies from past decades report that transient Ca^2+^ accumulation in astrocyte end‐feet provokes adjacent arteriole diameter dilatation or constriction via formation and release of vasoactive lipids.

#### 1.2.2 Astrocytic end‐feet and frataxin

**FIGURE 1:**
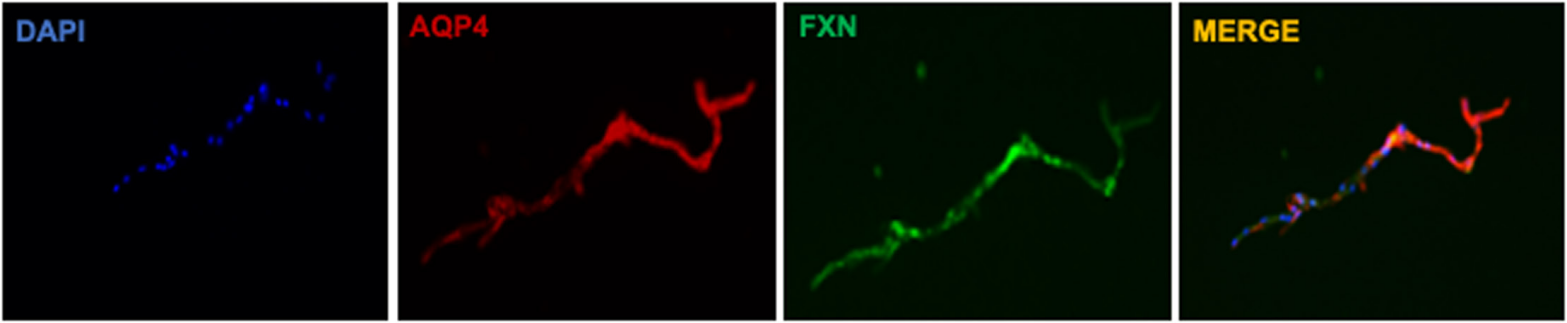
Double immunostaining of cerebral microvessels with the astrocyte end‐feet marker, aquaporin 4 (AQP4), and frataxin (FXN)

Astrocytic end‐feet surround the cerebral vasculature and regulate cerebral blood flow during ischemia, linking elevation of neuronal activity to elevation in blood flow.^70^ Astrocytic end‐feet are microdomains that contain highly metabolically active mitochondria that exhibit dynamic Ca^2+^ signaling.^71^, ^72^ In this study, we observed co‐expression of FXN and aquaporin‐4 (a marker of astrocyte end‐feet) in mouse cerebral microvessels (Figure 1). Given the role of FXN in mitochondrial function, FXN in astrocytic end‐feet may contribute to astrocytic Ca^2+^ signaling and cerebral blood flow regulation during ischemia. Direct observation of Ca^2+^ elevations in astrocytic end‐feet in vivo following neuronal synaptic activity evoked by physiological stimuli may provide important clues for determining how the neurovascular response is dynamically regulated in real time, and investigations into the role of astrocytic FXN in the neurovascular response may illuminate potential translational therapeutic strategies.

## 2 CONCLUSION

Herein, we have summarized the importance of astrocytic mitochondrial function and the astrocytic mitochondria protein FXN in ischemia‐induced changes in metabolism, mitochondrial dynamics, and cerebral blood flow. Moreover, we have discussed how the mitochondrial protein FXN, which is expressed in astrocyte end‐feet, may regulate cerebral blood flow and maintain oxidative balance. FXN is highly expressed in the mitochondria of both neurons and astroglia; however, compared to astrocytes, neurons express higher levels of FXN and are more sensitive to FXN deficiency, with complete absence of FXN leading to neuronal death. In addition, the half‐life of FXN is shorter in neurons than in astrocytes,^73^ suggesting that ischemic challenge may result into accelerated neuronal cell death compared to astrocytes. Hence, enhanced astrocytic FXN activity may incur better protective effects. However, the expression and function of FXN in astrocytes remain unclear. FXN silencing inhibits cell proliferation and induces cell death, leading to significant upregulation of the tumor suppressor protein p53, the cell cycle regulatory protein p21, and the apoptotic marker caspase‐3 in human astrocyte cultures.^74^ Further, FXN deficiency alters mitochondrial morphology and causes aberrant oxidative stress, neuronal, and astrocytic cell death.^75^ However, the mechanism by which FXN levels are regulated in astrocytes under physiological and pathological conditions and during ischemia is not fully understood. As FXN expression is increased during ischemic injury, and loss of astrocyte‐specific FXN expression leads to aberrant oxidative stress, therapies that maintain or increase FXN expression may be neuroprotective in patients experiencing ischemia. Specifically targeting astrocytic FXN may protect astrocytic mitochondria from damage and dysfunction to preserve and support the neuroprotective role of astrocytes in ischemia and prevent ischemic damage. Future studies are warranted to expand our understanding of FXN function and probe its therapeutic potential. Many questions remain unanswered, including (1) are ischemic changes following manipulations of mitochondrial proteins in astrocytes mediated by oxidative damage through glutamate and calcium; (2) how does the ischemic response alter mitochondrial structure and function within astrocytes; (3) what is the function of FXN within astrocytes; and (4) how does maintaining or increasing FXN expression alter ischemic injury? The evidence presented in this review demonstrates the importance of astrocytic mitochondrial functions and dynamics during ischemia, and provides a rationale to target these processes as therapies to control ischemic injuries.

## CONFLICT OF INTEREST

Dr. Xiaoming Hu is an Editorial Board member of CNS Neuroscience and Therapeutics and a co‐author of this article. To minimize bias, they were excluded from all editorial decision‐making related to the acceptance of this article for publication. No other conflicts of interests exist.

## Data Availability

The data that support the findings of this study are available from the corresponding author upon reasonable request.
